# Correlations between COVID-19 Case Growth and Mental Health-Related Internet Search: An Unexpected Finding

**DOI:** 10.18502/ijph.v49i10.4706

**Published:** 2020-10

**Authors:** Tenghao ZHANG

**Affiliations:** School of Business and Law, Edith Cowan University 270 Joondalup Drive Joondalup, WA 6027, Australia

## Dear Editor-in-Chief

At the time of writing, the number of global confirmed COVID-19 cases has topped 18.8 million with over 707,000 deaths (1). The Internet plays a pivotal role during this unprecedented pandemic (2,3) in the way that people predominantly use the Internet to contact each other and acquire information due to sweeping stay-at-home orders and strict lockdown restrictions imposed by authorities around the world. Health-related mental health issues could lead to spikes in online information search (4, 5). Therefore, this letter aims to investigate whether the rise of regional COVID cases is correlated with the increase in residents’ online searches of mental health-related information. An unexpected finding emerged from the analysis.

Specifically, I examined the correlation between new COVID-19 case growth and Google search interests in the 50 states and the capital of the United States. The US is the current epicentre of the pandemic which is accounted for over 26% of the world’s total confirmed cases. The number of new cases in the U.S is still growing fast as it added a million new cases in just 15 d as of late Jul 2020 (1). I retrieved the case number data by state from the U.S CDC authority (6) and then I divided the new reported cases in the past week (29th Jul to 4th Aug) by the total previous cases (before 29th Jul) and resulted in a case growth indicator (mean=11.1%, SD=7.63). The Internet search data were obtained from the Google Trends website. The website uses a normalized relative search volume (RSV) to measure the search interests on Google of a particular topic. The RSV can be restricted to a specific time period and administrative location such that comparisons can be made (7). Drawing on the WHO’s description of “mental disorders” (8) and the “related queries” feature in Google Trends, I retrieved the RSV data by U.S state (during 30th Jul to 5th Aug) of 14 mental health-related search terms which are considered to be closely linked to COVID-19. Table 1 reports the correlation coefficients between the regional case growth and RSVs. Among the 14 search terms, 10 are found to be negatively correlated to case growth at a significance level of 0.05, while only one term (distress) was positive but not significant (*P*>0.05). Disorder-related queries were the most negatively related to case growth, with “eating disorder” took the lead (*r*=−0.51, *P*<0.01). Other search terms such as anxiety, mental illness, insomnia were all negatively correlated to case growth (*r*< −0.3, *P*<0.05). The results are somewhat counterintuitive, as I initially expected those terms to be positively related to regional case growth. Although still not fully understood, I speculate that there are two possible explanations. First, when people are under stressful circumstances such as the deterioration of regional pandemic situation, they may be less likely to stay active on the Internet as usual. Second, a lagged effect could have existed between case growth and people’s mental health issues.

**Table 1:** Correlations between regional COVID-19 case growth and mental health-related Google search interests in the United States

***Search term***	***r***	***P***	***Search term***	***r***	***P***
Eating disorder	−0.51	0.00^**^	insomnia	−0.30	0.03^*^
Disorder	−0.45	0.00^**^	stress	−0.27	0.05^*^
Bipolar disorder	−0.42	0.00^**^	addiction	−0.26	0.05^*^
Autism	−0.35	0.01^**^	depression	−0.13	0.35
Anxiety	−0.33	0.02^*^	fear	−0.05	0.71
Psychiatry	−0.32	0.02^*^	panic	−0.04	0.78
mental illness	−0.31	0.03^*^	distress	0.23	0.10

* *P* < 0.05;

** *P* < 0.01 (two-tailed)

The recently fast-growing regions may actually be less affected during the last several months of the outbreak. However, I also analysed the correlations between RSVs and regional case severity (measured by total confirmed cases and total deaths per 100,000 population), positive correlations have still not been found. Therefore, the first explanation seems more plausible.
